# DNA analysis of soil extracts can be used to investigate fine root depth distribution of trees

**DOI:** 10.1093/aobpla/plu091

**Published:** 2015-02-02

**Authors:** Sean L. Bithell, Lucy T. T. Tran-Nguyen, Mark N. Hearnden, Diana M. Hartley

**Affiliations:** 1 Plant Industries, Northern Territory Department of Primary Industry and Fisheries, GPO Box 3000, Darwin, NT 0801, Australia; 2 CSIRO Ecosystem Sciences, GPO Box 1700, Canberra, ACT 2601, Australia; 3 Present address: New South Wales Department of Primary Industries, Tamworth Agricultural Institute, 4 Marsden Park Rd, Tamworth, NSW 2340, Australia

**Keywords:** DNA persistence, fine-roots, root DNA concentration, root DNA density (RDD)

## Abstract

Understanding the root distribution of trees by soil coring is time**-**consuming as it requires the separation of roots from soil and classification of roots into particular size classes. This labour-intensive process can limit sample throughput and therefore sampling intensity. We investigated the use of quantitative polymerase chain reaction (qPCR) on soil DNA extractions to determine live fine root DNA density (RDD, mg DNA m^−2^) for mango (*Mangifera indica*) trees. The specificity of the qPCR was tested against DNA extracted from 10 mango cultivars and 14 weed species. All mango cultivars and no weeds were detected. Mango DNA was successfully quantified from control soil spiked with mango roots and weed species. The DNA yield of mango root sections stored in moist soil at 23–28 °C declined after 15 days to low concentrations as roots decayed, indicating that dead root materials in moist soil would not cause false-positive results. To separate large roots from samples, a root separation method for field samples was used to target the root fragments remaining in sieved (minimum 2 mm aperture) soil for RDD comparisons. Using this method we compared the seasonal RDD values of fine roots for five mango rootstock cultivars in a field trial. The mean cultivar DNA yields by depth from root fragments in the sieved soil samples had the strongest relationship (adjusted multiple *R*^2^ = 0.9307, *P* < 0.001) with the dry matter (g m^−2^) of fine (diameter <0.64 mm) roots removed from the soil by sieving. This method provides a species-specific and rapid means of comparing the distribution and concentration of live fine roots of trees in orchards using soil samples up to 500 g.

## Abstract (precis)

Knowledge of a tree species or cultivar's fine root distribution is important. However, the time and resource requirements of established soil core based methods where live from dead root determination is required, act as a constraint to large studies. We developed a method to determine live fine root DNA density for mango (*Mangifera indica*). Soil-root samples had large roots separated by sieving (min. 2 mm aperture) and soil DNA extractions made on the sieved soil containing fine root fragments that had passed through the sieve. We showed that the DNA yields of these samples could determine fine root distribution.

## Introduction

Developing information on fine root and associated biomass distribution of trees is important to a number of fundamental research areas, including within season and annual growth cycles (Hendricks *et al.* 2006; Milchunas 2009); root longevity and carbon turnover (Eissenstat and Yanai 1997; Valverde-Barrantes *et al.* 2007; Goebel *et al.* 2011); and water or nutrient uptake (Green and Clothier 1999; Coehlo and Borges 2004; Morgan *et al.* 2007). Understanding of how different species or cultivars effect fine root growth (Hendrick and Pregitzer 1996), root longevity and turnover (Eissenstat and Yanai 1997; Valverde-Barrantes *et al.* 2007) and water or nutrient uptake (Morgan *et al.* 2007; da Silva *et al.* 2011) is also required.

To improve the productivity of horticultural tree crops, information on the root traits of rootstock cultivars associated with desirable breeding objectives such as vigour control, fruit size and yield efficiency is required (Gregory *et al.* 2013). For fruit tree crops, knowledge of the distribution and density of fine roots is also important to optimize the efficiency of irrigation and fertigation methods (Coehlo and Borges 2004; Morgan *et al.* 2007). As such there is a need for new methods that allow the rapid analysis of large numbers of field samples. In addition, it would be useful if techniques to study fine roots enable live roots to be distinguished from dead roots. Especially, as the proportion of live to dead roots and the rate of root decomposition can vary among species, as found for tropical tree species (Valverde-Barrantes *et al.* 2007). Therefore, methods that characterize the live fine root component of tropical trees could be more meaningful than methods for the total fine root component. Such techniques will also be useful to assess the impacts of tree root diseases (Graham *et al.* 2013).

Our study species was mango. In production regions such as the semi-arid environment of the Northern Territory (NT) of Australia, mango fruit production occurs in the dry season and irrigation is dependant on finite sources of groundwater for irrigation and fertigation (Crane *et al.* 1997; Pigram 2006).

In live root studies of other tree species, roots were removed from soil samples by wet sieving, then separated based on colour, texture or chemical staining (Persson 1990; Hendrick and Pregitzer 1996; Schroth 2003). The use of root colour for mango is difficult as new growth is white but becomes suberized and dark-brown to black in colour (Sharma and Sharma 1988). The window to assess washed roots before they start to decay is only 48 h for refrigerated samples (Valverde-Barrantes *et al.* 2007) or several weeks for samples stored at 1–2 °C (Persson 1990). Roots can be stored longer for microscopic assessments when stained and stored at 5 °C in 17 % acetic acid (Livesley *et al.* 1999). Using such methods, a single core can take 4–8 h to assess (Persson 1990). This time requirement is a significant constraint for large-scale field studies (Mackie-Dawson and Atkinson 1991; Schroth and Kolbe 1994; Schroth 2003).

Another method such as ground-penetrating radar is not suitable for studying fine roots, distinguishing roots of mixed species or separating live from dead roots (Nadezhdina and Cermak 2003; Butnor *et al.* 2008). An X-ray method detects fine roots, but includes combined measurement of live and dead roots (Pierret *et al.* 2005). In-growth core techniques record fine root production, such as a study of mango where core cavities were filled with commercial potting mix (Blaikie *et al.* 2004). However, artefacts in comparison to non-disturbed samples are found in some study systems with this method (Ostonen *et al.* 2005; Milchunas 2009). Minirhizotrons are widely used with visual assessments used to determine live roots from dead roots (Eissenstat and Yanai 1997; Johnson *et al.* 2001). Although the insertion of fluorescence genes has provided a means of detecting the roots of a target species (Faget *et al.* 2010), that method cannot be used to assess mature trees planted prior to this insertion-reliant technique being available.

A soil DNA extraction-root qPCR method to quantify root DNA density (RDD) provides several advantages for root studies. The high throughput soil extraction-qPCR technique, capable of processing large (300–500 g) and numerous (minimum 160 samples day^−1^) soil samples (Ophel-Keller *et al.* 2008), is critical for field trial sample processing. DNA methods enable the targeting of specific species in mixed species samples and qPCR methods provide the ability to detect small amounts (25 mg of dry root (kg soil)^−1^) of root material (Mommer *et al.* 2008; Riley *et al.* 2010; Haling *et al.* 2011). Traditionally wet washing accompanies root sorting (Persson 1990), but if the qPCR method uses soil DNA extractions there is no need to separate all root material from soil. This is advantageous as root condition degradation has been implicated by reduced DNA yields for the washing processes (Haling *et al.* 2012).

The RDD method provides a different way of studying root responses and is not equivalent to root dry weight or root length. As the DNA content of different aged roots, sizes or root morphologies can differ within grass and tree species (Fisk *et al.* 2010; Haling *et al.* 2011), RDD values will be specific to the root type, size and age of the material used. Soil DNA-based RDD methods require development to study the particular root classes of trees in field trials. In field studies of grasses and wheat, the DNA yields of undisturbed soil cores have been used to study the RDD of the entire root system (Haling *et al.* 2012; Huang *et al.* 2013). However, such methods cannot be used for trees where the objective is to compare specific root size classes as the DNA yields of large roots cannot be distinguished from those of smaller roots [e.g. mango roots >20 mm in diameter occur (Coehlo *et al.* 2001)]. Large structural tree roots also have a more irregular distribution compared with fine roots (Schroth 2003; Levillain *et al.* 2011). Removing the larger root classes from the soil samples is thus necessary to study the differences in the fine root systems of trees.

Prior work had shown that root DNA degrades in moist soil (Riley *et al.* 2010), but the effects of root storage in dried soil were unknown. It was also shown that different sieve sizes separate different root diameter size classes from soil samples (Livesley *et al.* 1999) and qPCR analyses of soil DNA extractions could be used to quantify root DNA in soil samples (Riley *et al.* 2010; Haling *et al.* 2011, 2012). Many studies employing molecular methods of mango cultivars have been undertaken to determine the relationships between cultivars and wild relatives (see Dillon *et al.* 2013 and references within); however, there are no universal primers or probes available to detect a wide range of mango cultivars. A universal probe/primer for mango was identified as challenging due to polymorphism among cultivars (Yonemori *et al.* 2002). The research questions investigated were: (i) can a probe/primer for detecting a wide range of mango cultivars be developed; (ii) what are the effects of dry storage of soil on root DNA and (iii) which root diameter size classes are represented by soil qPCR analyses of sieved soil samples containing root fragments. The results of these studies were used in unison to investigate mango fine root RDD using qPCR analyses of soil DNA extractions from sieved dried soil samples. Research focused on Kensington Pride (KP), which is the most commonly used rootstock cultivar in Australia.

## Methods

We developed a field-capable mango RDD method through a series of experiments. Following the development of a mango-specific probe, its specificity using qPCR was evaluated on DNA extracts of mango and weed tissue (Exp. 1), to compare the root DNA concentration of mango rootstock cultivars (Exp. 2). Other experiments were performed to evaluate the qPCR method on soil samples, including effects of soil type on root DNA yield (Exp. 3); root tissue concentration in soil and DNA yield relationship (Exp. 4); and root tissue degradation conditions on DNA yield (Exp. 5). The field capability for fine RDD quantification was trialled using sieved soil samples containing root fragments where we investigated the fine RDD of five mango rootstock cultivars in a field trial (Exp. 6).

### Soil and plant samples

#### Soil collections

Soil samples were collected from Darwin and Katherine, NT, Australia, at sites where mango had never been grown (control soils) and from under mango trees (mango soils) (Table 1). Samples were collected using hand augers (Dormer Soil Samplers, SA5010C, hole diameter 58 mm) and surface debris was removed before coring. Augers were thoroughly cleaned between the sampling of different trees and control areas. All soils were dried in forced air ovens and then stored in double-layered plastic bags in an air-conditioned facility.

**Table 1.** Listing of four soil sources from two localities, Berrimah Agricultural Research Centre, Darwin (D) and Fox Road^1^, Katherine (K) used in this study with land use history, plant species present at sampling and sample depth details. Label names including ‘Control’ indicate that mangoes had never been grown at those sites, label names including ‘Mango’ were sites where mangoes were growing and sampling occurred under mango trees. Experimental numbers (Exp. no.) also indicate which soil source was used in experiments.

Label and Exp. no.	Land use	Species present at sampling	Sample depths (cm)
D Field site Control, Exp. 3	Vegetables and pasture for >15 years	*Sorghum bicolor*, *Alysicarpus vaginealis*, *Macroptilium gracile*, *Chamaechrista rotundifolia*, *Stylosanthes humilis, Crotalaria goreensis, Oldenlandia corymbosa*, *Corchorus trilocularis*, *Boerhavia dominii*, *Sida acuta*, *Cyperus rotundus*, *Cynodon dactylon, Urochloa mosambicennsis*	0–180 in steps of 30
K Field site Control, Exps. 2, 4, 5	Forage cropping	*Centrosema pascuorum*, *Cenchrus echinatus*, *Brachiaria pubigera*, *Urochloa mosambicensis, Boerhavia dominii*	0–60
K Field site Mango 1, Exp. 3	Mango	Mango, KP rootstock	0–180 in steps of 30
K Field site Mango 2, Exp. 6	Mango	Mango, rootstock cultivars: Water tank (NT14), KP (NT16), Kurukan (NT21), MYP (NT50), Vellaikulamban (NT51). *Cajans geminatus*, *Tridax procumbens*, *Bracheria decumbens*, *Vigna lanceolata* var. *filiformus*, *Urochloa mosambicensis*	0–60 cm in steps of 15

^1^Mango trees at this site had paclobutrazol applied annually as a collar drench in late January or February at 20 mL (a.i. 250 g L^−1^) per tree.

Darwin soils were collected from Berrimah Agricultural Research Centre (BARC) [Lateritic Red Earth, Deep Red Petroferric Kandosol (Hill *et al.* 2011)]. Darwin control samples were collected from an area that had been used for vegetable trials and pasture for ∼15 years before a forage sorghum trial from December 2009 to April 2010. Two separate sets of samples from this site were used. The first set was prepared from samples collected in March 2010 (D Field site Control) (Table 1). Composite samples from duplicate cores for each 30 cm depth zone down to 1.8 m were prepared from soil dried at 65 °C for 48 h. Analyses of 0–15 cm samples from this site had soil total carbon of 1.91 % and pH (water method) of 5.48. The percentage of clay in the top 20 cm ranged from 29 to 34 %, while coarse sand ranged from 30 to 32 % and fine sand was 29 % (Hill *et al.* 2011).

Katherine soils were collected from Fox Road [Deep Red Magnesic Kandosol, Australian classification system (Hill *et al.* 2011)]. Control samples (K Field site Control) were collected in November 2010 from a field that had never been used for growing mango. Sampling in this field followed a transect at four 20-m intervals where four auger samples were collected from within a 2.5 m^2^ area. After the control soils were dried as previously described, soils were sieved (2 mm aperture) and rocky material removed, plant material collected in the sieve was returned to the sieved soil.

Mango soil samples were collected in September 2010, about 50 m from the control field in a mango rootstock trial planting. The first mango soil samples (K Field site Mango 1) were collected from neighbouring guard row trees (14-year-old KP scion on KP rootstock) surrounding the trial. These samples were collected and prepared as described for D Field site Control.

#### Plant samples for sequencing, qPCR development and specificity testing

Mango root tissue was sampled from 6- to 9-month-old potted seedlings held in the NT, Department of Primary Industry and Fisheries (DPIF), Plant Industries Mango rootstock collection (Table 2).

**Table 2.** Mango qPCR specificity test using DNA of 12 mango (*Mangifera indica*) cultivars and 14 weed species (Exp. 1). Details of plant species, cultivar (NT accession code), source, sample code and tissue type are shown. DNA at a concentration of 100 pg µL^−1^ was used and the results were expressed as detected (+) and undetected (−) at cycle 40.

Species	Cultivar	Source	Sample code	Tissue	Detection
*Mangifera indica*	KP (NT16)	DPIF^1^ Darwin, NT	3^S2^, 8^S^, 9b, 21a^S^	Root	+
*M. indica*	NDM (NT36)	K. Rayner, Katherine, NT	23a^S^	Root	+
*M. indica*	NDM	D. Hamilton, Howard Springs, NT	11^S^	Root	+
*M. indica*	NDM	Viet Ma, Lambells Lagoon, NT	SAR 5^3^	Root	+
*M. indica*	NDM	Viet Ma, Lambells Lagoon, NT	SAR 1^3^	Stem	+
*M. indica*	MYP (NT50)	DPIF Darwin, NT	16a^S^	Root	+
*M. indica*	B (NT63)	DPIF Darwin, NT	17a^S^	Root	+
*M. indica*	Chok Anan	DPIF Darwin, NT	18a^S^	Root	+
*M. indica*	Pancho (NT10)^4^	DPIF Darwin, NT	19a^S^	Root	^2^
*M. indica*	Chandrakaran (NT55)	DPIF Darwin, NT	20^S^	Root	+
*M. indica*	Vellaikulamban (NT51)	DPIF Darwin, NT	22a^S^	Root	+
*M. indica*	13/1 (NT52)	DPIF Darwin, NT	24a^S^	Root	+
*M. indica*	Brodie (NT9)	DPIF Darwin, NT	25a^S^	Root	+
*Dactyloctenium aegyptium*		Darwin, NT	35	Root	−
*Digitaria spp.*		Darwin, NT	36	Root	−
*Chloris inflata*		Darwin, NT	37	Root	−
*Pennisetum typhoides*	Katherine Pearl	Darwin, NT	30	Root	−
*Sorghum bicolor*	Jumbo	Landmark, Queensland	32	Root	−
*Melinis repens*		Darwin, NT	38	Root	−
*Chamaecrista rotundifolia*		Darwin, NT	39	Root	−
*Boerhavia dominii*		Darwin, NT	40	Root	−
*Cleome viscose*		Darwin, NT	41	Root	−
*Sida acuta*		Darwin, NT	42	Root	−
*Crotalaria goreenis*		Darwin, NT	43	Root	−
*Ludwigia parviflora*		Darwin, NT	44	Root	−
*Ludwigia* species		Darwin, NT	45	Root	−
*Eleusine indica*		Darwin, NT	46	Root	−
*Mitrocarpus hirtus*		Darwin, NT	47	Root	−

All mango cultivars in the table are polyembryonic.

^S^Samples sequenced.

^1^Department of Primary Industry and Fisheries (DPIF).

^2^Samples sequenced only.

^3^DNA of young root and stem collected from seed germinated under aseptic conditions.

^4^NT accession of cv. Red Haromanis.

Mango samples: Roots (∼2–6 mm diameter) were sampled and any damaged or discoloured material was omitted. All samples were surface sterilized with a KIMWIPES^®^ tissue pre-wetted with 70 % ethanol. Root samples were then cut in fine cross-sectional slices stored wrapped in KIMWIPES^®^ tissue over calcium chloride granules in plastic vials sealed with parafilm and stored at 4 °C until required for use. All root diameter measurements in this study were made with a digital calliper (Lufkin model 103192).

Weed root samples: The seed of weed species (Table 2) were sourced and sown into heat sterilized coarse river sand in 1 L pots. These were propagated on a raised table with no pot to soil contact in a covered nursery. Seedlings were harvested after 5–6 weeks and root samples were prepared and stored as described for the mango root samples.

#### DNA extractions

DNA extractions from root tissues were conducted using the DNeasy Plant Mini Kit (QIAGEN, Australia) according to the manufacturer's instructions. Crushed glass was used as an abrasive to assist with grinding the mango material. Preliminary extractions used both KP leaf and root materials for comparison.

#### Mango PCR and primer design

In total 13 mango samples (10 cultivars, including 2 accessions of cv. NDM) were sequenced (Table 2). Root samples used for sequencing had the epidermis removed. The extracted DNA samples were first subjected to PCR amplification targeting the ITS region (ITS1—5.8S ribosomal gene—ITS2), using the universal ITS 5 and ITS 4 primers (White *et al.* 1990). PCRs were conducted in a 50 µL mixture containing 1× ImmoMix Red with 3 mM MgCl_2_ (Bioline, Australia) and 0.2 µM of each primer and 5–10 ng of DNA template. The PCR conditions used were: initial enzyme activation of 95 °C for 10 min followed by 35 cycles of denaturing at 95 °C for 1 min, annealing at 56 °C for 1 min, extension at 72 °C for 1 min and final extension of 72 °C for 10 min. The PCR products were separated on a 1 % agarose/ethidium bromide gel using 1× TAE buffer and visualized under UV light.

The PCR products were purified using the QIAquick PCR purification kit (QIAGEN) according to the manufacturer's instructions, with final elution in 30 µL sterile distilled water, pH 7.3. DNA concentration was estimated using agarose gel electrophoresis with the low mass ladder (Life Technologies, Australia) as a reference. The products were sequenced using the Big Dye Terminator Mix (Bioscience North Australia, Darwin, NT). Nucleotide sequences were analysed and edited using Geneious Pro (Drummond *et al.* 2011). Multiple sequence alignment was conducted and included GenBank accession numbers for *Mangifera indica*, cv. Irwin AB071669 and cv. Nam Doc Mai (NDM) AB071672 (Yonemori *et al.* 2002). Primer3 program (part of Geneious package) was used to determine candidates for qPCR primers and probes.

Regions within the ITS region were analysed and conserved regions of the internal transcribed region were used to determine the mango-specific probe and primer sequences for the qPCR test. The probe–primer combination was required to cover a range of mango rootstock cultivars. Sequences were subjected to BlastN searches against the non-redundant nucleotide database (http://blast.ncbi.nlm.nih.gov/Blast.cgi?PROGRAM=blastn&BLAST_PROGRAMS=megaBlast&PAGE_TYPE=BlastSearch&SHOW_DEFAULTS=on&LINK_LOC=blasthome) to identify species of highest similarity. The nucleotide sequence of cv. NDM (Hamilton) was compared with cv. NDM, GenBank Accession Number AB071672 (Yonemori *et al.* 2002). This sample was used as a reference for comparison with previously un-sequenced mango rootstocks.

#### Mango qPCR specificity test (Exp. 1)

This experiment evaluated the specificity of the probe–primer combination. Following preliminary studies on the development of probes and primers for qPCR, we developed a mango-specific probe–primer F1 (5′-TCGAGTCTTTGAACGCAAGTTG-3′) and R3 (5′-CCACGCCGAAAGATCGTT-3′) primers, and probe (6FAM-5′-ACACCCAGGCAGACGT-3′). The specificity of this probe–primer was trialled using qPCR testing on DNA from the roots of a wide range of mango cultivars and from roots of common tropical weed species (Table 2). DNA from roots of cv. NDM (Viet Ma) seed, sprouted under laboratory conditions in sterile media, was used to prepare a reference standard and to establish a standard curve for the qPCR. The mango qPCR was performed on a 7900HT real-time PCR system using TaqMan^®^ MGB (Minor Groove Binder) probe (Applied Biosystems, CA, USA) and QuantiTect Probe PCR Master Mix (QIAGEN GmbH, Hilden, Germany). PCRs were conducted in a 10 µL mixture containing 1× QuantiTect Probe Master Mix, 0.4 µM of each primer, 0.2 µM of probe and 0.4 ng of DNA template. The PCR conditions used were: step 1, 50 °C for 2 min; step 2, 95 °C for 15 min; step 3, 95 °C for 15 s; step 4, 60 °C for 1 min; steps 3–4 were repeated for 45 cycles.

#### Quantification of mango root DNA (Exp. 2)

This experiment compared the root DNA concentrations of mango rootstock cultivars. DNA was extracted from root tissue (minimum diameter range 1.1–2.8 mm, maximum diameter range 1.7–4.2 mm before epidermis was removed) from five cultivars (Watertank, KP, Kurakan, MYP and Vellaikulamban, Table 2). For all cultivars except Watertank, samples were prepared separately from each of the three 5- to 6-month-old potted seedlings. Kurakan, KP, MYP and Vellaikulamban seedlings were propagated from seeds collected from the same mother trees used as seed sources for the rootstock field trial. The original mother tree of NT14 had died; therefore, roots for NT14 were collected from a single tree which was planted as a nucellar seedling from the original mother tree. Root sample preparation followed the methods described for mango samples used in the sequencing and specificity sections. DNA was extracted using the DNeasy Plant Mini Kit (QIAGEN) from ∼500 mg samples of root tissue, to provide three root extracts per cultivar. Neat DNA extracts were diluted to 200 pg µL^−1^ and used for qPCR. The cycle threshold (Ct) value, the number of cycles for the fluorescent signal to cross the threshold, was determined for all samples. The Ct values were then converted to mg mango DNA per mg dry root.

#### Quantification of mango DNA in soil

Four separate batches of dried soil samples (300–500 g) from five experiments (Exps. 3–7) were shipped from Darwin to Adelaide for DNA testing by a commercial service, Root Disease Testing Service (RDTS, South Australian Research and Development Institute (SARDI), Adelaide, Australia) (Ophel-Keller *et al.* 2008). Each batch of samples included three batch check soil samples (300 g K Field site Control) without amendment and another three with 100 mg kg^−1^ of KP (NT16) root added. These batch checks were prepared in Darwin and shipped to RDTS with each batch.

To determine the presence of PCR inhibitors in the soil extracts, a known amount of an exogenous organism (internal control) was included in each sample by SARDI before extraction. Lower amplification of this internal control indicates that the extraction has a poor DNA recovery or contains PCR inhibitors (Ophel-Keller *et al.* 2008). Work by Haling *et al*. (2011) showed that DNA extraction efficiency, measured by the internal control, was similar across soil types and rarely affected by soil inhibitors.

All DNA values from soil qPCR analysis were provided as pg DNA per g soil. For field samples the total pg value of the entire sample was calculated from the total sample weight of soil and converted to m^2^ values as described for soil carbon by McKenzie *et al*. (2000) and expressed as mg DNA m^−2^ for each sample depth.

#### Soil type effects (Exp. 3)

This experiment evaluated the effects of soil type on DNA recovery and presence of PCR inhibitors in soil extracts. Samples of two soil types commonly used for mango production in the NT (D Field site Control and K Field site Mango 1) were used. The soil samples (300 g) were spiked with known amount of the internal control before DNA extraction as described previously.

#### Mango root concentration: DNA yield and cultivar detection in control soil (Exp. 4)

This experiment evaluated the qPCR quantification of root tissue in soil samples. Ten each of 1-month-old first- and second-order KP (NT16) potted seedlings were placed in an automated mist house on a raised metal grid for 8–12 weeks. Roots grew freely through the bottom of the pots and grid apertures, and also remained moist due to misting. Sliced root tissue (minimum diameter range 1.1–1.8 mm, maximum diameter range 3.2–4.4 mm) was dried at 40 °C for 48 h before being used to spike control (K Field site Control) soil samples. Soil samples (300 g per sample) were spiked with different concentrations of the dried aerially produced KP root slices (0, 25, 50, 100, 300, 800, 1600 mg kg^−1^ of soil) with four replicates of each concentration.

Root tissue from mango cultivars or weed species (Table 4) was sampled from potted plants, prepared and dried as described previously. This material was used to spike (300 mg root per kg soil) duplicate control soil samples (K Field site Control, 300 g per sample) for each of the mango cultivars or weed species.

#### Effects of root tissue degradation on DNA yield (Exp. 5)

This experiment evaluated the effects of root tissue degradation and soil conditions on DNA yield. Moderate (sectioned) and severe (sliced) root tissue disturbance treatments were compared for tissue stored in moist and dry soil over time. Aerially produced roots (as described in Exp. 4) from three potted 1-year-old NDM (Hamilton) seedlings were harvested and divided into three diameter classes: 1.5–2, 1–1.5 and 0.8–0.9 mm, and each class was divided into two equal wet weight groups. Sub-samples from each of the three size classes were then dried at 40 °C for 48 h. The average dry matter (DM) content across the size classes was 5.5 %. Half of each group was cut into 10 mm sections and the other half was finely sliced. A factorial experiment included three treatments: (i) soil moisture: dry soil (0.46 % gravimetric moisture content) or watered soil [to 75 % of field capacity (FC), 9.3 %] every 2–3 days; (ii) root fragment size: 10 mm root sections or finely cut slices of root; and (iii) time of sampling: Time 0, sampled at experimental setup; Time 1, after 2 weeks; and Time 2, after 4 weeks. A randomized complete block design with four replicates totalling 48 experimental units was used. Sectioned or sliced roots (220 mg wet weight, equivalent to DM concentration of 129 mg (kg soil)^−1^) were added to plastic bags containing 300 g of K Field site Control soil and mixed thoroughly. Distilled water was then added to the FC treatments to bring the sample to 75 % of FC. FC treatment bags were not sealed, dry treatments were sealed. Units for Time 1 and Time 2 were placed in an incubator (14 h 23 °C, 10 h 28 °C). Units from Time 0 were transferred to paper bags following setup and dried at 40 °C for 48 h before sealing in plastic bags for DNA analysis. FC treatments were removed and weighed every 2–3 days (average 48 h moisture loss 0.14 %, range 0.06–0.39 %) and distilled water added to a 75 % FC weight. Units for Time 1 and Time 2 were removed at 15 and 30 days, respectively, dried in paper bags, prepared for DNA analysis as described for Time 0.

### Field study

#### Rootstock cultivar field trial (Exp. 6)

This experiment evaluated the mango DNA yields of sieved root-removed soil samples of five rootstock cultivars in the field trial, for three sample depths and two time points. Mango soil samples were collected from under selected polyembryonic rootstock cultivar treatments in 11–12 November 2010 and 22–23 February 2011 from a mango rootstock trial in Katherine (K Field site Mango 2) (Table 1). The trial (five replicates of a balanced lattice design) was planted in 1996, and trees were spaced at 9 m between rows and 5 m within rows. Orchard management practices were intensive and semi-mechanized (Crane *et al.* 1997; Bally 2011). By 2010, the trees in the trial had filled their allocated space and were pruned post-harvest each year to maintain separate trees rather than a hedgerow system.

Soil-root samples were collected from five replicates of rootstock cultivars from five cultivar treatments (KP, MYP, Kurukan, Watertank and Vellaikulamban). Six cores were collected 0.5 m from the trunk of each tree using a sampling template providing equally spaced sampling positions. For the February sample, the template was rotated to position cores 0.3 m from the November 2010 positions. The six samples for each depth (0–15, 15–30 and 30–45 cm) were bulked providing a sample volume of 2378 cm^3^ per 15 cm depth per tree. Weed species up to a distance of 1.5 m from the trunk of each tree were recorded at sampling. The soil samples were dried (40 °C for 48 h) within 2.5 h of collection. Before sieving-root sorting and mango DNA analyses, the average soil moisture values from three selected samples were 0.47 and 0.48 %, respectively.

After drying, each sample was successively sieved through 8, 4 and 2 mm sieves (Endecott, London, UK). The sieving took a maximum of 15 min per sample. Roots were removed at each stage of sieving, and the root-removed sieved soil sorted for root fragments 2 mm or greater in diameter or length and these were removed. For the November samples, roots were then shaken in a 0.23 mm sieve to remove adhering sand and soil then sorted by eye on a white background. Sorting was aided by comparison with manufactured cylinders 0.64, 1.88 and 7.5 mm diameter. Roots were placed into four size categories of <0.64, 0.64–1.88, >1.88–7.5 and >7.5 mm. Root size sorting took ca. 1–2 h per sample. These root samples were dried (65 °C for 48 h) and weighed.

Following sieving and root removal, each soil sample was then sub-sampled for DNA analysis. To provide a representative proportion, a sub-sample representing 15 % of the weight of total sample was sub-sampled in a 70 mm deep tray using a level surface sub-sampling method described by Schroth (2003). The qPCR analysis of these soil samples for mango DNA was carried out as described in Exp. 2.

Root and root fragment removed sieved soil samples from one sampled tree per block (*n* = 5) were pooled for analyses from each of the 0–15, 15–30, 30–45 cm zones and an additional depth zone of 45–60 cm. Total carbon decreased from 0.52 to 0.08 % with increasing depth, the percentage of clay in samples increased with depth from 5.0 to 17.6 %, coarse sands decreased with depth from 79.1 to 67.4 %, fine sands increased with depth from 10.9 to 14.0 %.

Storage time effects on the analyses of field samples were investigated using all KP soil samples from the November collection (*n* = 15), these had qPCR analyses made on two separate sub-samples, analysed at 6 weeks interval. Samples were stored after sieving and root removal had been carried out in double-layered plastic bags in an air-conditioned facility.

### Analyses

Analysis of variance (ANOVA) was used to compare cultivar effects on root Ct and DNA concentrations (Exp. 2). The relationships between (i) root DM soil concentration and mango DNA yields for KP was examined with linear regression for origin constrained to zero (Exp. 4) and (ii) soil pg DNA values and root DM values from the rootstock trial was examined with linear regression (non-constrained) (Exp. 6). ANOVA was used for analysis of the following factors; tissue treatment, soil moisture, assessment time and their interactions in the DNA persistence experiment (Exp. 5). For the rootstock study (Exp. 6), weed densities were examined for their effect on soil DNA values by comparing trees with and without weeds. A generalized linear model with a Gaussian link function was used, with individual models for each depth, separate repeated-measure models were used for the November and February data. Comparison of soil DNA values for storage time effects in the rootstock study was made using a repeated-measures ANOVA for time effects and the factor sample depth, block was included. Comparison of soil DNA values at post-wet season and post-harvest periods for cultivars the rootstock study was made using a repeated-measures ANOVA model for the factors such as time and sample depth, including block. Tukey's HSD method was used for post-hoc pairwise comparisons where required. Analyses were carried out with S-PLUS (Insightful Corporation); for more detailed information on analyses **[see Supporting Information]**.

## Results

### Cultivar sequencing

A multiple sequence alignment of the 113 bp qPCR target region showed that there was sequence variation between the mango cultivars studied. In total, there were six positions with variability. One variation was located on the Mango F1 primer, and a second in the Mango R3 primer and the remainder was found in the conserved areas. There was no sequence variability found in the probe region **[see Supporting Information]**.

### Plant tissue-only experiments

#### Mango qPCR specificity test (Exp. 1)

The mango qPCR was specific and sensitive. It detected all the mango DNA tested (average Ct of 23.8 for 100 pg µL^−1^ mango DNA) but not the weed species (Table 2).

#### Quantification of mango root DNA (Exp. 2)

The Ct values differed between cultivars, whereby KP and Vellaikulamban were significantly lower than Kurakan and MYP. The Ct of Watertank was also lower than that of MYP (Table 3). The mango root DNA concentration differed significantly; the cv. KP had higher concentrations than the four other cultivars.

**Table 3.** Determination of Ct values (with standard deviations, std. dev.) and root DNA concentrations (ng mango DNA mg^−1^ dry root) for root samples of five mango cultivars (Exp. 2), df = 10 for all comparisons, standard error of the mean (SEM) and Tukey’s HSD (HSD) values also presented.

Cultivar	Ct (std. dev.)	Root DNA of mango (ng mango DNA per mg dry root)
Watertank (NT14)	22.89 (0.28)	0.873
KP (NT16)	22.22 (0.26)	3.965
Kurukan (NT21)	23.50 (0.19)	0.858
MYP (NT50)	24.14 (0.35)	0.576
Vellaikulamban (NT51)	22.31 (0.34)	1.199
SEM	0.168	0.4646
HSD	0.783	2.0767

### Experiments with soil

For the soil DNA analysis, all negative batch check samples included had values below the detection limit (5 pg (g soil)^−1^) with a range of 0–2.9 pg (g soil)^−1^. For the positive batch check samples, the average value was 798.7 pg (g soil)^−1^ (range 624.1–1029.6).

#### Soil type effects (Exp. 3)

The concentrations of the internal control DNA extracted from soil samples from the two sites and various depths were similar [D Field site Control, average 758 641, range 179 424 (fg well^−1^) and K Field site Mango 1, average 681 072, range 88 757 (fg well^−1^)].

#### Mango root concentration: DNA yield and cultivar detection in control soil (Exp. 4)

**Figure 1.**
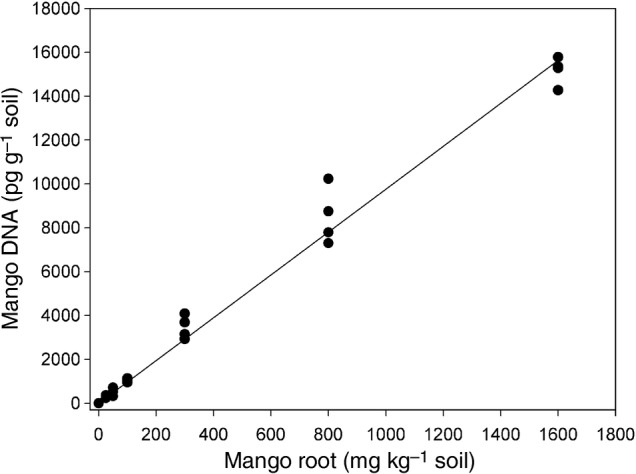
Root dry matter concentrations (0, 25, 50, 100, 300, 800 and 1600 mg kg^−1^) of KP root tissue in control soil plotted against pg DNA (g soil)^−1^ values (Exp. 4), with a fitted linear regression, *y* = 9.7666 (*x*), *P* < 0.001, adjusted multiple *R*^2^ = 0.9909, standard error of coefficient 0.1769, df = 27 (Exp. 4).

Control soil spiked with KP root concentrations from 25 to 1600 mg kg^−1^ had a significant linear relationship with mango DNA yields (Fig. 1). The soil qPCR method also detected samples of other polyembryonic cultivars (MYP, B, Chok Anan, Pancho, Chandrakaran, Vellaikulamban, 13/1 and Brodie) or source of NDM (NT36), and a monoembryonic cultivar (Irwin) (Table 4). Picogram values among cultivars were variable, the minimum value of which was 367 pg DNA (g soil)^−1^ across all cultivars.

**Table 4.** Quantification of mango root DNA (pg g^−1^ soil) in soil spiked with root material of each of 11 mango cultivars and six weed species (Exp. 4), mango cultivars (NT accession code). Source details of root material except for cv. Irwin is provided in Table 2.

Species	Cultivars	Root DNA (pg g^−1^ soil)
*Mangifera indica*	KP (NT16)	1270.9
*M. indica*	NDM (NT36)	2646.7
*M. indica*	MYP (NT50)	367.5
*M. indica*	B (NT63)	3244.3
*M. indica*	Chok Anan	1221.5
*M. indica*	Pancho (NT10)	516.1
*M. indica*	Chandrakaran (NT55)	977.4
*M. indica*	Vellaikulamban (NT51)	1606.3
*M. indica*	13/1 (NT52)	1318.8
*M. indica*	Brodie (NT9)	844.0
*M. indica*	Irwin^1^	1589.3
*Digitaria spp.*	–	0.0
*Chloris inflata*	–	0.0
*Pennisetum typhoides*	Katherine Pearl	0.0
*Sorghum bicolor*	Jumbo	0.0
*Melinis repens*	–	0.0
*Crotalaria goreenis*	–	0.0

^1^The only monoembryonic cultivar included in the study.

#### Effects of root tissue degradation on DNA yield (Exp. 5)

**Figure 2.**
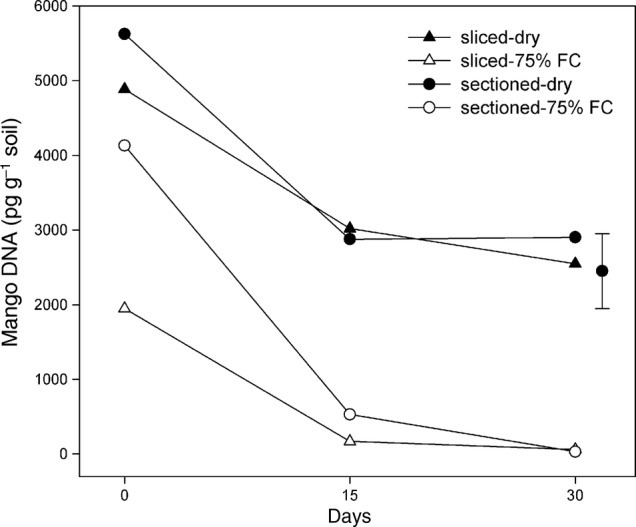
Results of a mango root DNA (pg DNA g^−1^ soil) persistence experiment (Exp. 5) over 30 days for NDM (Hamilton) root tissue subjected to four treatments: sliced root in dry soil; sliced root in 75 % FC soil; 10 mm root sectioned in dry soil; 10 mm root sectioned in 75 % FC soil, df = 33, standard error of the mean = 208.1, Tukey’s HSD = 998.8.

There was a significant three-way interaction (*P* = 0.014) between the tissue disturbance treatment, soil moisture and assessment time (Fig. 2). The pg g^−1^ values for the sliced-75 % FC treatment were significantly lower at Day 0 in comparison to the three other treatments. The sectioned-75 % FC also had significantly less DNA than the sectioned-dry treatment. At Day 15 the sectioned-75 % FC had similar pg values to the sliced-75 % FC treatment, with large differences between the soil moisture treatments. The trend for large soil moisture effects continued to Day 30 when both the pg values for the 75 % FC treatments were approaching zero with final values of 60.3 pg for sliced-75 % FC and 30.2 pg for sectioned-75 % FC. In contrast, the average value of the two dry treatments was >2700 pg.

#### Rootstock cultivar field trial (Exp. 6)

Weed species were present in the rootstock field trial. Twelve and nine of the 25 trees had weed seedlings present in November and February, respectively. Weed densities were low with ranges of 0.14–1.84 and 0.14–2.12 weeds m^−2^, respectively. In November, five weed species were present, and in February, a sixth species (*Urochloa mosambicensis*) was also present (Table 1, K Field site Mango 2). There were no significant differences (*P* = 0.353–0.910) in soil mango DNA concentrations for November or February samples at any of the sample depths between trees with and without weeds present **[see Supporting Information]**.

**Figure 3.**
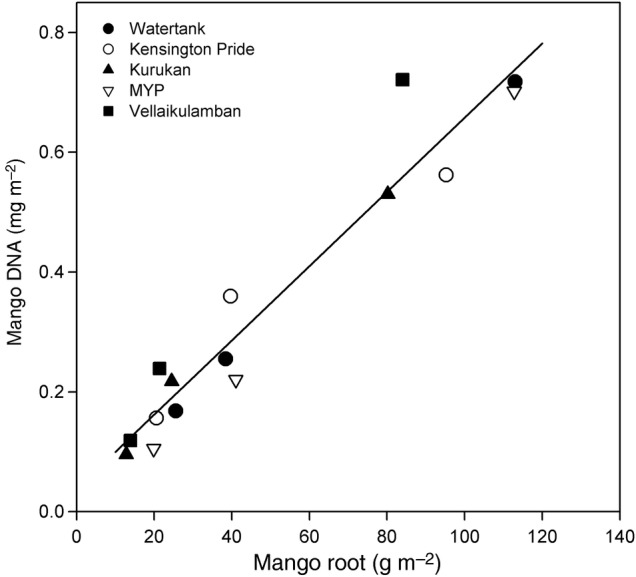
Regression results of average Class 1 (diameter <0.64 mm) mango root dry matter (DM g m^−2^) values for samples of five rootstock cultivars (see legend) sampled in November from three sample depths (0–15, 15–30 and 30–45 cm) in the rootstock trial (Exp. 6) between average mango DNA concentrations in soil (mg m^−2^) of sieved soil after root removal with fitted regression, *y* = 0.0062*x* + 0.0376.

For the comparison of pg DNA values from sieved soil samples collected in November and the weight of roots removed from these samples, the mean mango DNA (dependant variable) values for each cultivar by sample depth had the strongest relationship (adjusted multiple *R*^2^ = 0.9307, *P* < 0.001, df = 13) with the DM of Class 1 roots (Fig. 3). The relationship between the larger three root classes and total root DM was poor (multiple *R*^2^ range 0.0022–0.2463, *P* > 0.05 in all cases).

Soil samples collected in November for rootstock KP (NT16, *n* = 15) had a separate analysis of duplicate sub-samples made 6 weeks after the first analyses. A repeated-measures analysis for mean mango DNA values gave no significant time effect (*P* = 0.940) or time by depth interaction effect (*P* = 0.460) between the two dates.

**Figure 4.**
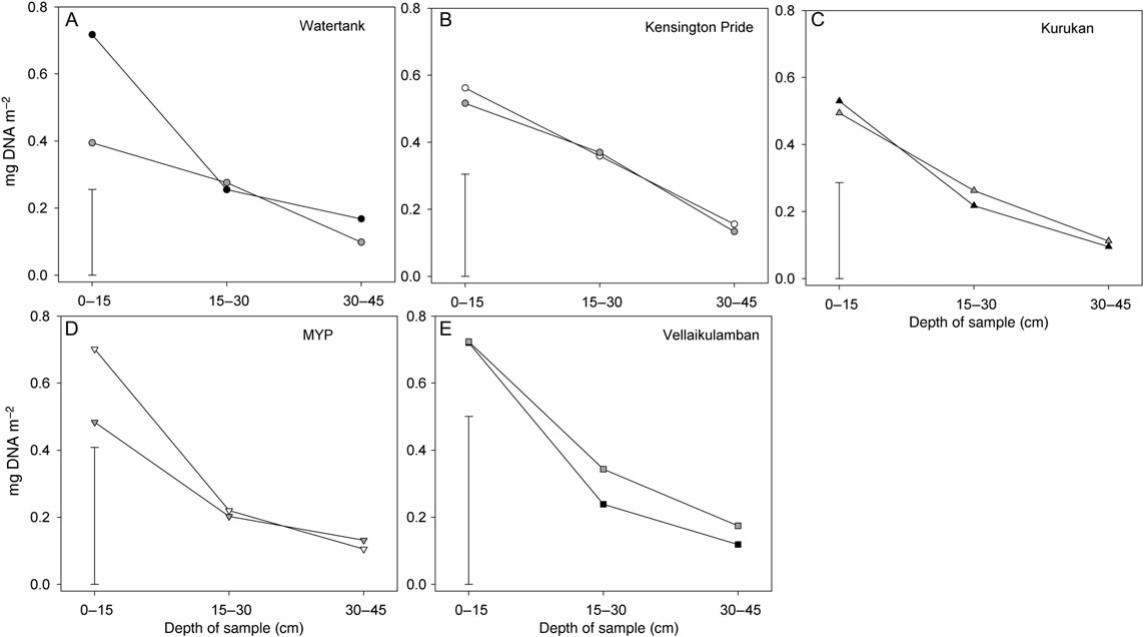
Mango DNA (mg DNA m^−2^) of sieved soil for the five cultivars (plots A–E) for three sample depths sampled in November (post-harvest) and the following February (post-wet season) in the rootstock trial (Exp. 6), df = 12 for each comparisons, HSD values shown by vertical bars.

For the separate analysis of soil DNA concentrations from November and February samples for each cultivar, cv. Watertank had a significant date by depth interaction (*P* = 0.035, tHSD = 0.2559). For cv. Watertank the November sample (0.717 mg DNA m^−2^) had higher concentrations than the February sample (0.395 mg DNA m^−2^) for the 0–15 cm depth (Fig. 4). For all cultivars depth effects were significant (*P* < 0.05), including significantly higher DNA values in the 0–15 than the 15–30 cm zone, except for cv. KP which only had a higher DNA concentration in the 0–15 than the 30–45 cm zone.

## Discussion

We developed a RDD method that detected the root DNA of all mango rootstock cultivars tested. We showed that the method could quantify depth and site differences in RDD values for fine roots for the predominate Australian rootstock cultivar, KP (Crane *et al.* 1997). Further field testing showed that the method also had potential for rootstock cultivar comparisons.

The root DNA persistence experiment (Exp. 5) demonstrated that the contribution of DNA from dead roots after 15–30 days in moist soil was minimal (Fig. 2). Riley *et al*. (2010) also found that the DNA concentrations of lyophilized *Lolium perenne* and *Trifolium subterraneum* roots in moist soil declined to low concentrations over a 7-day period. In our study, moist soil conditions also assisted initial DNA degradation, as at Time 0, a greater degradation of the finely sliced root material in moist soil than finely sliced root material in dry soil occurred. The DNA degradation results for a moist soil environment indicated that the method was suitable for sampling irrigated trees or for sampling during the wet (monsoonal) season.

To provide a focus on the DNA of fine roots and effects of an uneven distribution of larger roots on DNA yields, the soil samples were dried and progressively passed through smaller sieves (min 2 mm aperture). The soil DNA analysis was carried out on the sieved soil that had passed through all sieves. The mango DNA concentration in the sieved soil was strongly related but not equal to the DM of the finest root class removed by the smallest root class in sieves (Fig. 3). Although there were substantial DM values for other root classes (data not presented), the poor relationship of the soil DNA yield with the DM of larger root classes confirmed that the DNA present in the root material was not associated with larger root classes.

To address tree root distribution heterogeneity issues, our method required analyses of large soil samples. High levels of sub-sampling and replication are often required in root studies due to uneven root distribution (Mackie-Dawson and Atkinson 1991; Schroth 2003), but such requirements may act as a sampling intensity limitation. Root size sorting (1–2 h per sample) was required to establish which size class of root the fragments remaining in sieved soil were associated with (Fig. 3); however, this more time-consuming root size sorting would not be required in further experiments under the same conditions.

The method was also used to compare the seasonal RDD of mango rootstock cultivars. One cultivar, Watertank, had greater DNA concentrations at the post-harvest sample than the post-wet season sample (Fig. 4). This indicated that cv. Watertank could, for example, have a greater mass of live fine roots during the fruit production period. A trial that included the five same rootstock cultivars grafted to KP (Fig. 3) found that cv. Watertank had higher cumulative fruit yields than the four other rootstock cultivars (Smith *et al.* 2008). In India, non-grafted mango trees had individual preflowering and post-harvest fine (<1.5 mm) root densities that were positively associated with individual tree fruit yield across three biennial bearing mango cultivars (Rao and Mukherjee 1988). Our RDD findings for cv. Watertank grafted to KP [an erratic non-alternate bearing scion (de Souza *et al.* 2004)] showed rootstock cultivar differences in seasonal root traits, rather than associations with the on–off bearing patterns of individual trees for non-grafted alternate bearing cultivars (Rao and Mukherjee 1988). Further work would be required to establish which root parameter (root age, root mass etc.) was responsible for the RDD finding and how this may relate to productivity. Importantly, the RDD method provided a rapid means of identifying cultivar root traits associated with productivity from which specific hypothesis could be investigated in future work.

Differences were detected in mango root tissue DNA concentrations between five rootstock cultivars. Although we targeted a conserved region to design the qPCR test, a multiple sequence alignment of the target region of 10 cultivars showed six positions with sequence variability **[see** **Supporting Information]**. As already stated, the DNA contents from different age roots, sizes or root morphologies can differ within species (Fisk *et al.* 2010; Haling *et al.* 2011), therefore some differences in root morphology or age among mango cultivars could have contributed to DNA concentration differences. Ct values among cultivars can vary through variable sequences or single-nucleotide polymorphisms within a sequence (Wu *et al.* 2010). Intraspecific polymorphism and sites of nucleotide additivity in the ITS region do occur among mango cultivars (Yonemori *et al.* 2002). To ensure these sequence differences and potential differences in the copy number of the reference genes do not artificially create cultivar differences. A reference single copy qPCR assay will need to be developed as outlined in Huang *et al*. (2013) for cultivar to cultivar comparisons. Because of this potential influence, we adopted a conservative approach of only comparing between timepoints for each cultivar in our field study of rootstock cultivars rather than among cultivars.

Some studies have investigated the root distribution of individual mango trees (Avilan and Meneses 1979; Coehlo *et al.* 2001; de Almeida *et al.* 2009). These studies have focused on the entire root system making interpretations about the size range of fine roots of mango difficult to interpret. However, results from one study indicate that roots <0.5 mm in diameter occur at low incidence (Coehlo *et al.* 2001). Our method was developed to focus on the fine root fragments of mango, for species with finer diameter roots the method may have to be adapted using smaller aperture sieves. Our method was also developed on high sand content soils. For plant species occurring on soil types that have high clay contents and bind heavily when dried (e.g. Vertosols), the sieving of soil to separate roots may not be easily possible.

## Conclusions

We have developed a method to study the differences in distribution and density of fine root systems of the cv. KP using soil samples from mango orchards. The advantages of this method include: a sensitive and species-specific qPCR assay that is not affected by dead roots in moist soil; allows rapid pre-processing by using sieved soil samples (300–500 g) to remove the larger roots that would have increased between sample variation; and is suitable for a high throughput. The method also shows potential for detecting differences in cultivar RDD values.

## Accession Numbers

Nucleotide sequences were submitted to GenBank and were assigned the following accession numbers. Kensington Pride NT16 (8) KJ833763; Nam Doc Mai NT36 (23a) KJ833765; MYP NT50 (16a) KJ833764; B NT63 (17a) KJ833759; Chok Anan (18a) KJ833762; Pancho NT10 (19a) KJ833766; Chandrakaran NT55 (20) KJ833761; Vellaikulamban NT51 (22a) KJ833767; 13/1 NT52 (24a) KJ833758 and Brodie NT9 (25a) KJ833760.

## Sources of Funding

Our work was funded by the Northern Territory Research & Innovation Fund, Northern Territory Government, Australia.

## Contributions by the Authors

S.L.B. and L.T.T.T-N. initiated and obtained funding for the conceptual qPCR mango root detection method and the resulting experimental path. They carried out all preliminary plant tissue-only DNA and initial probe–primer development work, worked on all experiments and co-wrote this manuscript. S.L.B. conducted the field studies, collected all material (soil and roots) for the experiments and conducted preliminary statistical analysis. L.T.T.T-N. developed an initial range of probes and primers. D.M.H. developed the final version of the probe and primer. M.N.H. contributed to the design of all experiments and carried out all statistical analyses.

## Conflicts of Interest Statement

None declared.

## Supporting Information

The following Supporting Information is available in the online version of this article –

**Figure S1.** Provides a multiple sequence alignment for root tissue samples from ten mango cultivars depicting the targeted ITS region (ITS1-5.8S rRNA-ITS2) used in the qPCR test.

**File S2.** Provides a statistical summary of experiments including ANOVA and regression tables.

Additional Information
