# Supplementary material for: DNA analysis of soil extracts can be used to investigate fine root depth distribution of trees
Source: AoB Plants. 2015 Feb 2;7:plu091. doi: 10.1093/aobpla/plu091 (PMC4313792; doi:10.1093/aobpla/plu091)
Supplement: Additional Information [file supp_7_plu091_index.html]

DNA analysis of soil extracts can be used to investigate fine root depth distribution of trees — DNA analysis of soil extracts can be used to investigate fine root depth distribution of trees — Additional Information 

# DNA analysis of soil extracts can be used to investigate fine root depth distribution of trees

## Additional Information

Additional Information

**Files in this Data Supplement:**

- Supporting Information - Docx file
