# Supplementary material for: DNA analysis of soil extracts can be used to investigate fine root depth distribution of trees
Source: AoB Plants. 2015 Feb 2;7:plu091. doi: 10.1093/aobpla/plu091 (PMC4313792; doi:10.1093/aobpla/plu091)
Supplement: Additional Information [file supp_plu091_plu091supp.docx]

**Supporting information 1:**

Figure 1. Multiple sequence alignment for root tissue samples from ten mango cultivars depicting the targeted ITS region used in the qPCR test. For full names of the abbreviated mango cultivars with NT accession codes refer to Table 2. The sample numbers in this figure are shown in brackets. *Mango NDM refers to GenBank Accession Number AB071672. Nucleotide Y can be C/T, M (A/C) and K (G/T). Multiple samples of the cultivars KP and NDM are presented.


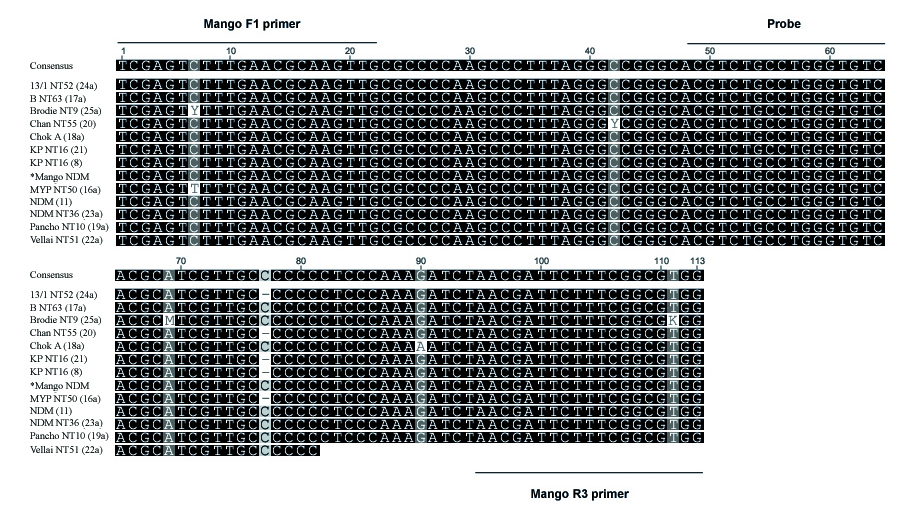


**Supporting Information 2, Anova and regression tables**

**Experiment 2**. Comparison of mean adjusted cycle threshold values and mean root DNA concentrations values (micrograms DNA/mg dry root *10^6^) for five NT cultivars. Means were compared with a single Factor ANOVA model, and pairwise comparisons were analysed using the Tukey method.

Table Exp. 2.1. ANOVA results for comparison of mean adjusted cycle threshold values.

| **Effect** | **DF** | **Sum of Squares** | **Mean Square** | **F** | **P** |
| --- | --- | --- | --- | --- | --- |
| Cultivar | 4 | 7.91 | 1.98 | 23.33 | <0.001 |
| Residual | 10 | 0.85 | 0.08 |  |  |

Table Exp. 2.2. ANOVA results for comparison of mean root DNA concentration values (micrograms DNA/mg dry root *10^6^).

| **Effect** | **DF** | **Sum of Squares** | **Mean Square** | **F** | **P** |
| --- | --- | --- | --- | --- | --- |
| Cultivar | 4 | 23.48 | 5.87 | 9.06 | 0.002 |
| Residual | 10 | 6.48 | 0.65 |  |  |

**EXP 4, Soil cv. KP concentration and DNA yield**

> summary(model.noint)

Call: lm(formula = F1Ay ~ F1Ax - 1)

Residuals:

Min     1Q Median    3Q  Max

-1354 -11.05   35.7 175.3 2417

Coefficients:

Value Std. Error t value Pr(>|t|)

F1Ax 9.7666 0.1769 55.2226 0.0000

Residual standard error: 642.9 on 27 degrees of freedom

Multiple R-Squared: 0.9912      Adjusted R-squared: 0.9909

F-statistic: 3050 on 1 and 27 degrees of freedom, the p-value is 0

**EXP 5, root DNA persistence**

**Model for time1**

Df Sum of Sq Mean Sq F Value Pr(F)

block 3 1191764 397255 1.49297 0.2813981

tissue 1 8547876 8547876 32.12475 0.0003066

moisture 1 19615377 19615377 73.71879 0.0000125

tissue:moisture 1 2082610 2082610 7.82689 0.0207920

Residuals 9 2394754 266084

Means for Tissue (moistures pooled)

95 % simultaneous confidence intervals for specified

linear combinations, by the Sidak method

critical point: 2.6567

response variable: pg.DNA.g.soil

intervals excluding 0 are flagged by '****'

Estimate Std.Error Lower Bound Upper Bound

cut 3420 182 2930 3900 ****

whole 4880 182 4390 5360 ****

Means for Moisture (tissues pooled)

95 % simultaneous confidence intervals for specified

linear combinations, by the Sidak method

critical point: 2.6567

response variable: pg.DNA.g.soil

intervals excluding 0 are flagged by '****'

Estimate Std.Error Lower Bound Upper Bound

FC 3040 182 2550 3520 ****

dry 5250 182 4770 5740 ****

Means and comparisons for Tissue between Moistures (dry=adj1,FC=adj2)

95 % simultaneous confidence intervals for specified

linear combinations, by the Sidak method

critical point: 3.0463

response variable: pg.DNA.g.soil

intervals excluding 0 are flagged by '****'

Estimate Std.Error Lower Bound Upper Bound

cut.adj1 1950 258 1160 2730 ****

whole.adj1 4130 258 3350 4920 ****

cut.adj2 4880 258 4100 5670 ****

whole.adj2 5620 258 4840 6410 ****

95 % simultaneous confidence intervals for specified

linear combinations, by the Dunnett method

critical point: 2.6567

response variable: pg.DNA.g.soil

intervals excluding 0 are flagged by '****'

Estimate Std.Error Lower Bound Upper Bound

cut.adj1-whole.adj1 -2180 365 -3150 -1210 ****

cut.adj2-whole.adj2 -740 365 -1710 229

Means and comparisons between Moistures within Tissue (cut=adj1,whole=adj2)

95 % simultaneous confidence intervals for specified

linear combinations, by the Sidak method

critical point: 3.0463

response variable: pg.DNA.g.soil

intervals excluding 0 are flagged by '****'

Estimate Std.Error Lower Bound Upper Bound

FC.adj1 1950 258 1160 2730 ****

dry.adj1 4880 258 4100 5670 ****

FC.adj2 4130 258 3350 4920 ****

dry.adj2 5620 258 4840 6410 ****

95 % simultaneous confidence intervals for specified

linear combinations, by the Dunnett method

critical point: 2.6567

response variable: pg.DNA.g.soil

intervals excluding 0 are flagged by '****'

Estimate Std.Error Lower Bound Upper Bound

FC.adj1-dry.adj1 -2940 365 -3910 -1970 ****

FC.adj2-dry.adj2 -1490 365 -2460 -524 ****

**Model for time2**

Df Sum of Sq Mean Sq F Value Pr(F)

block 3 1053695 351232 2.8195 0.0995271

tissue 1 47863 47863 0.3842 0.5507221

moisture 1 26987765 26987765 216.6466 0.0000001

tissue:moisture 1 254344 254344 2.0418 0.1868064

Residuals 9 1121134 124570

Means for Tissue (moistures pooled)

95 % simultaneous confidence intervals for specified

linear combinations, by the Sidak method

critical point: 2.6567

response variable: pg.DNA.g.soil

intervals excluding 0 are flagged by '****'

Estimate Std.Error Lower Bound Upper Bound

cut 1590 125 1260 1920 ****

whole 1700 125 1370 2030 ****

Means for Moisture (tissues pooled)

95 % simultaneous confidence intervals for specified

linear combinations, by the Sidak method

critical point: 2.6567

response variable: pg.DNA.g.soil

intervals excluding 0 are flagged by '****'

Estimate Std.Error Lower Bound Upper Bound

FC 349 125 17.8 681 ****

dry 2950 125 2620.0 3280 ****

Means and comparisons for Tissue between Moistures (dry=adj1,FC=adj2)

95 % simultaneous confidence intervals for specified

linear combinations, by the Sidak method

critical point: 3.0463

response variable: pg.DNA.g.soil

intervals excluding 0 are flagged by '****'

Estimate Std.Error Lower Bound Upper Bound

cut.adj1 169 176 -369.00 706

whole.adj1 530 176 -7.47 1070

cut.adj2 3020 176 2480.00 3560 ****

whole.adj2 2880 176 2340.00 3410 ****

95 % simultaneous confidence intervals for specified

linear combinations, by the Dunnett method

critical point: 2.6567

response variable: pg.DNA.g.soil

intervals excluding 0 are flagged by '****'

Estimate Std.Error Lower Bound Upper Bound

cut.adj1-whole.adj1 -362 250 -1020 301

cut.adj2-whole.adj2 143 250 -520 806

Means and comparisons between Moistures within Tissue (cut=adj1,whole=adj2)

95 % simultaneous confidence intervals for specified

linear combinations, by the Sidak method

critical point: 3.0463

response variable: pg.DNA.g.soil

intervals excluding 0 are flagged by '****'

Estimate Std.Error Lower Bound Upper Bound

FC.adj1 169 176 -369.00 706

dry.adj1 3020 176 2480.00 3560 ****

FC.adj2 530 176 -7.47 1070

dry.adj2 2880 176 2340.00 3410 ****

95 % simultaneous confidence intervals for specified

linear combinations, by the Dunnett method

critical point: 2.6567

response variable: pg.DNA.g.soil

intervals excluding 0 are flagged by '****'

Estimate Std.Error Lower Bound Upper Bound

FC.adj1-dry.adj1 -2850 250 -3510 -2190 ****

FC.adj2-dry.adj2 -2350 250 -3010 -1680 ****

**Model for time3**

Df Sum of Sq Mean Sq F Value Pr(F)

block 3 134450 44817 1.1881 0.3679469

tissue 1 104491 104491 2.7701 0.1303961

moisture 1 28726384 28726384 761.5590 0.0000000

tissue:moisture 1 146919 146919 3.8949 0.0798834

Residuals 9 339484 37720

Means for Tissue (moistures pooled)

95 % simultaneous confidence intervals for specified

linear combinations, by the Sidak method

critical point: 2.6567

response variable: pg.DNA.g.soil

intervals excluding 0 are flagged by '****'

Estimate Std.Error Lower Bound Upper Bound

cut 1300 68.7 1120 1490 ****

whole 1470 68.7 1280 1650 ****

Means for Moisture (tissues pooled)

95 % simultaneous confidence intervals for specified

linear combinations, by the Sidak method

critical point: 2.6567

response variable: pg.DNA.g.soil

intervals excluding 0 are flagged by '****'

Estimate Std.Error Lower Bound Upper Bound

FC 45.3 68.7 -137 228

dry 2730.0 68.7 2540 2910 ****

Means and comparisons for Tissue between Moistures (dry=adj1,FC=adj2)

95 % simultaneous confidence intervals for specified

linear combinations, by the Sidak method

critical point: 3.0463

response variable: pg.DNA.g.soil

intervals excluding 0 are flagged by '****'

Estimate Std.Error Lower Bound Upper Bound

cut.adj1 60.3 97.1 -236 356

whole.adj1 30.3 97.1 -266 326

cut.adj2 2550.0 97.1 2250 2840 ****

whole.adj2 2900.0 97.1 2610 3200 ****

95 % simultaneous confidence intervals for specified

linear combinations, by the Dunnett method

critical point: 2.6567

response variable: pg.DNA.g.soil

intervals excluding 0 are flagged by '****'

Estimate Std.Error Lower Bound Upper Bound

cut.adj1-whole.adj1 30 137 -335 395.0

cut.adj2-whole.adj2 -353 137 -718 11.6

Means and comparisons between Moistures within Tissue (cut=adj1,whole=adj2)

95 % simultaneous confidence intervals for specified

linear combinations, by the Sidak method

critical point: 3.0463

response variable: pg.DNA.g.soil

intervals excluding 0 are flagged by '****'

Estimate Std.Error Lower Bound Upper Bound

FC.adj1 60.3 97.1 -236 356

dry.adj1 2550.0 97.1 2250 2840 ****

FC.adj2 30.3 97.1 -266 326

dry.adj2 2900.0 97.1 2610 3200 ****

95 % simultaneous confidence intervals for specified

linear combinations, by the Dunnett method

critical point: 2.6567

response variable: pg.DNA.g.soil

intervals excluding 0 are flagged by '****'

Estimate Std.Error Lower Bound Upper Bound

FC.adj1-dry.adj1 -2490 137 -2850 -2120 ****

FC.adj2-dry.adj2 -2870 137 -3240 -2510 ****

**EXP 6, rootstock field trial experiment**

**a) Analysis for weed effects on mango DNA results**

Methods: Data for trees with and without volunteer seedlings and data for trees with and without weeds were compared separately using a generalized linear model, with individual models for each depth. Separate models were used for the November and February data.

Results: There were no significant differences in soil mango DNA concentrations for November samples at any of the sample depths between trees with and without mango volunteers present (Table X). For the February Volunteer data, only one tree had a volunteer so no comparisons were possible.

Similarly, there were no significant differences in soil mango DNA concentrations for November or February samples at any of the sample depths between trees with and without weeds present (Table Y).

Table X. Mango soil DNA concentrations (mean mg mango DNA/mg dry root with SE) for trees with and without mango volunteers for November and February samples. P-values are for the test that means in the two groups are similar.

| Month | Depth | Volunteers Absent | | Volunteers Present | | P |
| --- | --- | --- | --- | --- | --- | --- |
|  |  | mean | SE | mean | SE |  |
| November | 0-15 | 0.632 | 0.0822 | 0.662 | 0.1006 | 0.9952 |
|  | 15-30 | 0.305 | 0.0585 | 0.199 | 0.0478 | 0.1905 |
|  | 30-45 | 0.137 | 0.0209 | 0.118 | 0.0224 | 0.5313 |
| February | 0-15 | 0.497 | 0.0406 | no data | | - |
|  | 15-30 | 0.293 | 0.0348 |  |  | - |
|  | 30-45 | 0.133 | 0.0170 |  |  | - |

Table Y. Mango soil DNA concentrations (mean mg mango DNA/mg dry root with SE) for trees with and without weeds for November and February samples. P-values are for the test that means in the two groups are similar.

| Month | Depth | Weeds Absent | | Weeds Present | | P |
| --- | --- | --- | --- | --- | --- | --- |
|  |  | mean | SE | mean | SE |  |
| November | 0-15 | 0.632 | 0.0827 | 0.662 | 0.0981 | 0.8116 |
|  | 15-30 | 0.278 | 0.0627 | 0.237 | 0.0492 | 0.6132 |
|  | 30-45 | 0.131 | 0.0203 | 0.126 | 0.0234 | 0.8939 |
| February | 0-15 | 0.489 | 0.0523 | 0.582 | 0.0918 | 0.3533 |
|  | 15-30 | 0.285 | 0.0458 | 0.301 | 0.0481 | 0.8217 |
|  | 30-45 | 0.129 | 0.0215 | 0.133 | 0.0272 | 0.9102 |

Depth 0-15cm

Analysis of Deviance Table

Gaussian model

Response: data$mg.dna.m2

Terms added sequentially (first to last)

Df Deviance Resid. Df Resid. Dev F Value Pr(F)

NULL 12 1.323596

data$Tridax 1 0.003550541 11 1.320045 0.02958683 0.8665548

means se

Absent 0.7249016 0.1181333

Present 0.6909321 0.1640941

Depth 15-30cm

Analysis of Deviance Table

Gaussian model

Response: data$mg.dna.m2

Terms added sequentially (first to last)

Df Deviance Resid. Df Resid. Dev F Value Pr(F)

NULL 12 0.6161306

data$Tridax 1 0.01147064 11 0.6046599 0.2086743 0.6566971

means se

Absent 0.3358333 0.09580616

Present 0.2747763 0.06732235

Depth 30-45cm

Analysis of Deviance Table

Gaussian model

Response: data$mg.dna.m2

Terms added sequentially (first to last)

Df Deviance Resid. Df Resid. Dev F Value Pr(F)

NULL 12 0.06329235

data$Tridax 1 0.0002860619 11 0.06300628 0.04994233 0.8272597

means se

Absent 0.1545013 0.02677045

Present 0.1641434 0.03381825

**b) Regressions of dry matter values for roots extracted by sieving (4 size classes and total dry matter) vs. sieved soil DNA values**

Summary Table: Multiple R^2^ and significance (P) of linear regression of the dry matter of roots from Classes 1 to 4 (g root/m^2^) and average mango DNA (mg DNA/m^2^) concentrations for the five rootstocks sampled in 2010 across three depth zones, df = 13.

| Root diameter classes | Adjusted Multiple R^2^ | P |
| --- | --- | --- |
| Class 1, < 0.64 mm | 0.9307 | < 0.0001 |
| Class 2, 0.64 to 1.88 mm | 0.2463 | 0.0599 |
| Class 3, >1.88-7.5 mm | 0.0022 | 0.8690 |
| Class 4, > 7.5 mm | 0.2459 | 0.0601 |
| Total | 0.1463 | 0.1593 |

Model for Class 1 (nb Class 5=total)

Call: lm(formula = mg.dna.m2 ~ class)

Residuals:

Min 1Q Median 3Q Max

-0.07164 -0.03116 -0.02023 0.01203 0.1633

Coefficients:

Value Std. Error t value Pr(>|t|)

(Intercept) 0.0376 0.0286 1.3180 0.2103

class 0.0062 0.0005 13.2158 0.0000

Residual standard error: 0.0644 on 13 degrees of freedom

Multiple R-Squared: 0.9307 Adjusted R-squared: 0.9254

F-statistic: 174.7 on 1 and 13 degrees of freedom, the p-value is 6.514e-009

Model for Class 2 (nb Class 5=total)

Call: lm(formula = mg.dna.m2 ~ class)

Residuals:

Min 1Q Median 3Q Max

-0.2809 -0.1298 -0.0721 0.1254 0.4047

Coefficients:

Value Std. Error t value Pr(>|t|)

(Intercept) 0.0752 0.1416 0.5308 0.6045

class 0.0142 0.0069 2.0613 0.0599

Residual standard error: 0.2124 on 13 degrees of freedom

Multiple R-Squared: 0.2463 Adjusted R-squared: 0.1884

F-statistic: 4.249 on 1 and 13 degrees of freedom, the p-value is 0.05987

Model for Class 3 (nb Class 5=total)

Call: lm(formula = mg.dna.m2 ~ class)

Residuals:

Min 1Q Median 3Q Max

-0.2579 -0.1911 -0.1071 0.1998 0.3663

Coefficients:

Value Std. Error t value Pr(>|t|)

(Intercept) 0.3684 0.1563 2.3574 0.0347

class -0.0005 0.0031 -0.1682 0.8690

Residual standard error: 0.2444 on 13 degrees of freedom

Multiple R-Squared: 0.002172 Adjusted R-squared: -0.07458

F-statistic: 0.0283 on 1 and 13 degrees of freedom, the p-value is 0.869

Model for Class 4 (nb Class 5=total)

Call: lm(formula = mg.dna.m2 ~ class)

Residuals:

Min 1Q Median 3Q Max

-0.3198 -0.1675 -0.04721 0.166 0.2931

Coefficients:

Value Std. Error t value Pr(>|t|)

(Intercept) 0.4540 0.0765 5.9385 0.0000

class -0.0004 0.0002 -2.0590 0.0601

Residual standard error: 0.2125 on 13 degrees of freedom

Multiple R-Squared: 0.2459 Adjusted R-squared: 0.1879

F-statistic: 4.239 on 1 and 13 degrees of freedom, the p-value is 0.06011

Model for Class 5 (nb Class 5=total)

Call: lm(formula = mg.dna.m2 ~ class)

Residuals:

Min 1Q Median 3Q Max

-0.3227 -0.1653 -0.04371 0.1661 0.3255

Coefficients:

Value Std. Error t value Pr(>|t|)

(Intercept) 0.4698 0.1023 4.5929 0.0005

class -0.0003 0.0002 -1.4929 0.1593

Residual standard error: 0.2261 on 13 degrees of freedom

Multiple R-Squared: 0.1463 Adjusted R-squared: 0.08068

F-statistic: 2.229 on 1 and 13 degrees of freedom, the p-value is 0.1593

**c) Nov vs Feb soil DNA values using individual model each cultivar, block included**

Depth x Month BY cv

Model for NT14 KRS tank (values below means marked [1] are HSD’s)

Error: depth:Block

Df Sum of Sq Mean Sq F Value Pr(F)

depth 2 0.9361100 0.4680550 16.13007 0.0003972014

Residuals 12 0.3482105 0.0290175

Error: Within

Df Sum of Sq Mean Sq F Value Pr(F)

Month 1 0.1149024 0.1149024 6.520687 0.02529933

Month:depth 2 0.1586545 0.0793273 4.501807 0.03477949

Residuals 12 0.2114544 0.0176212

D.00.15 D.15.30 D.30.45

0.5561163 0.2654356 0.1332061

[1] 0.2322355

Post.Dry Post.Wet

0.3801403 0.256365

[1] 0.1477646

Post.Dry Post.Wet

D.00.15 0.7174514 0.39478121

D.15.30 0.2548976 0.27597363

D.30.45 0.1680719 0.09834028

[1] 0.2559358

Model for NT16 KP

Error: depth:Block

Df Sum of Sq Mean Sq F Value Pr(F)

depth 2 0.7800554 0.3900277 5.778862 0.01746978

Residuals 12 0.8099056 0.0674921

Error: Within

Df Sum of Sq Mean Sq F Value Pr(F)

Month 1 0.0027051 0.00270507 0.1079498 0.7481530

Month:depth 2 0.0039866 0.00199329 0.0795454 0.9240189

Residuals 12 0.3007026 0.02505855

D.00.15 D.15.30 D.30.45

0.5391225 0.3644253 0.1449857

[1] 0.3541806

Post.Dry Post.Wet

0.3590069 0.3400154

[1] 0.17621

Post.Dry Post.Wet

D.00.15 0.5619172 0.5163277

D.15.30 0.3591056 0.3697450

D.30.45 0.1559978 0.1339736

[1] 0.3052047

Model for NT21 Kurukan

Error: depth:Block

Df Sum of Sq Mean Sq F Value Pr(F)

depth 2 0.8645842 0.4322921 14.71612 0.0005902785

Residuals 12 0.3525049 0.0293754

Error: Within

Df Sum of Sq Mean Sq F Value Pr(F)

Month 1 0.0005758 0.00057584 0.0260415 0.8744840

Month:depth 2 0.0083483 0.00417416 0.1887699 0.8303888

Residuals 12 0.2653493 0.02211244

D.00.15 D.15.30 D.30.45

0.5121063 0.2401175 0.1037074

[1] 0.2336631

Post.Dry Post.Wet

0.2809292 0.2896916

[1] 0.1655278

Post.Dry Post.Wet

D.00.15 0.52988064 0.4943319

D.15.30 0.21763966 0.2625954

D.30.45 0.09526736 0.1121474

[1] 0.2867025

Model for NT50 Mylepania

Error: depth:Block

Df Sum of Sq Mean Sq F Value Pr(F)

depth 2 1.263012 0.6315058 13.81629 0.0007705008

Residuals 12 0.548488 0.0457073

Error: Within

Df Sum of Sq Mean Sq F Value Pr(F)

Month 1 0.0361222 0.03612217 0.8062260 0.3868993

Month:depth 2 0.0851722 0.04258610 0.9504972 0.4138207

Residuals 12 0.5376483 0.04480402

D.00.15 D.15.30 D.30.45

0.5926919 0.211444 0.1184497

[1] 0.2914682

Post.Dry Post.Wet

0.3422282 0.2728288

[1] 0.2356194

Post.Dry Post.Wet

D.00.15 0.7016672 0.4837165

D.15.30 0.2200042 0.2028837

D.30.45 0.1050133 0.1318861

[1] 0.4081048

Model for NT51 Vellaikulamban

Error: depth:Block

Df Sum of Sq Mean Sq F Value Pr(F)

depth 2 1.794775 0.8973874 14.82612 0.0005718177

Residuals 12 0.726330 0.0605275

Error: Within

Df Sum of Sq Mean Sq F Value Pr(F)

Month 1 0.0219977 0.02199769 0.3260234 0.5785466

Month:depth 2 0.0130333 0.00651665 0.0965819 0.9086341

Residuals 12 0.8096728 0.06747273

D.00.15 D.15.30 D.30.45

0.7223345 0.2911469 0.1464989

[1] 0.3354088

Post.Dry Post.Wet

0.3595814 0.4137388

[1] 0.2891457

Post.Dry Post.Wet

D.00.15 0.7212017 0.7234673

D.15.30 0.2389803 0.3433135

D.30.45 0.1185621 0.1744356

[1] 0.5008151

**d) Storage time effects for soil DNA values**

Repeated measures (within effects) ANOVA to assess the effect of depth over time.

There is a significant difference by depth (p=0.035) but the interaction is not significant (p=0.460) meaning there is no significant difference in the mean amount of DNA by depth between time 1 and time 2.

Error: block:depth

Df Sum of Sq Mean Sq F Value Pr(F)

depth 2 0.982075 0.4910375 4.472006 0.03537759

Residuals 12 1.317630 0.1098025

Error: Within

Df Sum of Sq Mean Sq F Value Pr(F)

batch 1 0.0000523 0.000052298 0.0057977 0.9405602

batch:depth 2 0.0149662 0.007483120 0.8295683 0.4597778

Residuals 12 0.1082460 0.009020499

Means

Batch2 Batch4

D.00.15 0.561916 0.623996

D.15.30 0.359106 0.313494

D.30.45 0.156000 0.147454

upper 95%CL

Batch2 Batch4

D.00.15 0.7270205 0.7948512

D.15.30 0.5708557 0.4948579

D.30.45 0.1938166 0.1896772

lower 95%CL

Batch2 Batch4

D.00.15 0.3968115 0.4531408

D.15.30 0.1473563 0.1321301

D.30.45 0.1181834 0.1052308
